# Machine Learning‐Guided Discovery of Novel Inhibitors of *Escherichia coli* MurB

**DOI:** 10.1111/cbdd.70422

**Published:** 2026-11-05

**Authors:** Shuhua Song, Qingxin Liu, Aoqi Luo, Xiao Shi, Xinxin Si

**Affiliations:** ^1^ School of Pharmacy Jiangsu Ocean University Lianyungang China

**Keywords:** machine learning, molecular docking, MurB, virtual screening

## Abstract

MurB, a key enzyme involved in bacterial cell wall biosynthesis, represents a promising target for antibacterial drug discovery. In this study, a machine learning assay was employed to identify potential inhibitors targeting *E. coli* MurB. Five machine learning models were constructed using AutoMolDesigner, of which the RDKit_2D*‐*based Model_2 was selected as the optimal activity*‐*prediction model for virtual screening of an in*‐*house compound library, yielding 238 compounds. These compounds were subsequently subjected to FRED molecular docking, and 16 candidate compounds were selected based on their hydrogen*‐*bond interactions with the key active*‐*site residue ASN A:233. Preliminary enzyme inhibition assays conducted at 50 μM identified 4 compounds (Q9, Q11, Q12, and Q13) with inhibition rates exceeding 50%. Further IC₅₀ determination revealed values of 34.44, 31.45, 25.08, and 25.94 μM, respectively. Molecular dynamics simulations combined with principal component analysis were performed to investigate the dynamic behaviors and binding stability of the MurB*‐*ligand complexes. In addition, a CCK*‐*8 assay was conducted in HepG2 cells to evaluate the cytotoxicity of the active compounds preliminarily. Collectively, 4 low*‐*cytotoxic micromolar MurB inhibitors were identified in this study, offering promising scaffolds for further mechanistic investigation and structural optimization.

## Graphical abstract

Four *E. coli* MurB inhibitors (Q9, Q11, Q12, and Q13; IC₅₀: 25.08–34.44 μM) via machine learning‐based virtual screening, molecular docking, in vitro assays, and MD simulations, validating an efficient discovery strategy.

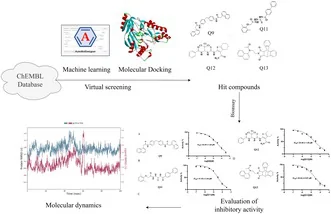

## 1 Introduction

Bacterial infections remain one of the leading causes of mortality worldwide. In 2019, diseases associated with bacterial infections were responsible for approximately 7.7 million deaths, accounting for 13.6% of all global fatalities (GBD 2019 Antimicrobial Resistance Collaborators 2022). *Escherichia coli* ( *E. coli* ) is one of the most prevalent Gram*‐*negative pathogens and is responsible for a wide range of infections (Mueller et al. 2026), including urinary tract infections (Mahesh et al. 2024), bloodstream infections (Gunasekaran et al. 2026), and intra*‐*abdominal infections (García‐Laorden et al. 2017). The increasing emergence of antibiotic*‐*resistant *E. coli* strains has posed a substantial challenge to existing therapeutic options (Kasanga et al. 2024; Nasrollahian et al. 2024). Therefore, identifying novel antibacterial targets and developing new inhibitors against *E. coli* are of considerable clinical and scientific importance.

The bacterial cell wall is essential for maintaining cellular integrity, promoting growth, and ensuring survival, and it is frequently utilized as a target in antimicrobial research (Fam et al. 2026). Peptidoglycan (PG), the primary structural component of the bacterial cell wall, accounts for approximately 50%*–*80% of its dry weight and plays a crucial role in sustaining mechanical strength and morphological stability (Gautam et al. 2011). PG biosynthesis is a highly conserved yet complex process that involves approximately 20 enzymatic reactions (Garde et al. 2021). Based on the subcellular localization of these reactions, the biosynthetic pathway can be divided into three stages (Scheffers and Pinho 2005; van Heijenoort 1998): the cytoplasmic stage, the membrane*‐*associated stage, and the extracytoplasmic stage. Each stage involves multiple essential enzymes that have been widely recognized as attractive targets for antibacterial drug development (Abrahams and Besra 2018).

MurB is a key cytoplasmic enzyme involved in the early stage of PG biosynthesis (Haque et al. 2023). It catalyzes the NADPH*‐*dependent reduction of UDP*‐*N*‐*acetylglucosamine enolpyruvate (UNAGEP) to UDP*‐*N*‐*acetylmuramic acid (UDP*‐*MurNAc), which is an essential precursor for bacterial cell wall assembly (Vollmer et al. 2008). Because this reaction is indispensable for the generation of fundamental building blocks of the bacterial cell wall, inhibition of MurB effectively disrupts PG biosynthesis, leading to impaired cell wall formation and suppression of bacterial growth (Acebrón‐García‐de‐Eulate et al. 2022). Consequently, MurB has been recognized as a highly promising antibacterial target, and in*‐*depth investigation of this enzyme may provide new strategies for combating bacterial infections.

To date, no MurB*‐*specific inhibitors have been approved for clinical use, and related research remains at an early exploratory stage. Only a limited number of MurB inhibitors have been reported (Mehta et al. 2023), which can be broadly classified into several chemical categories (Figure 1), including 4*‐*thiazolidinone derivatives (Andres et al. 2000), imidazolinone derivatives (Gilbert et al. 2006), pyrazolidinedione analogs, and phenylthiazolyl urea compounds (Francisco et al. 2004). Although these compounds exhibit measurable inhibitory activity against MurB in vitro, their structural diversity, antibacterial potency, drug*‐*likeness, and safety profiles remain limited and require further optimization (El Zoeiby et al. 2003). These limitations highlight the continued need for systematic screening and mechanistic studies aimed at identifying novel MurB inhibitor scaffolds (Zhou et al. 2022).

**FIGURE 1:**
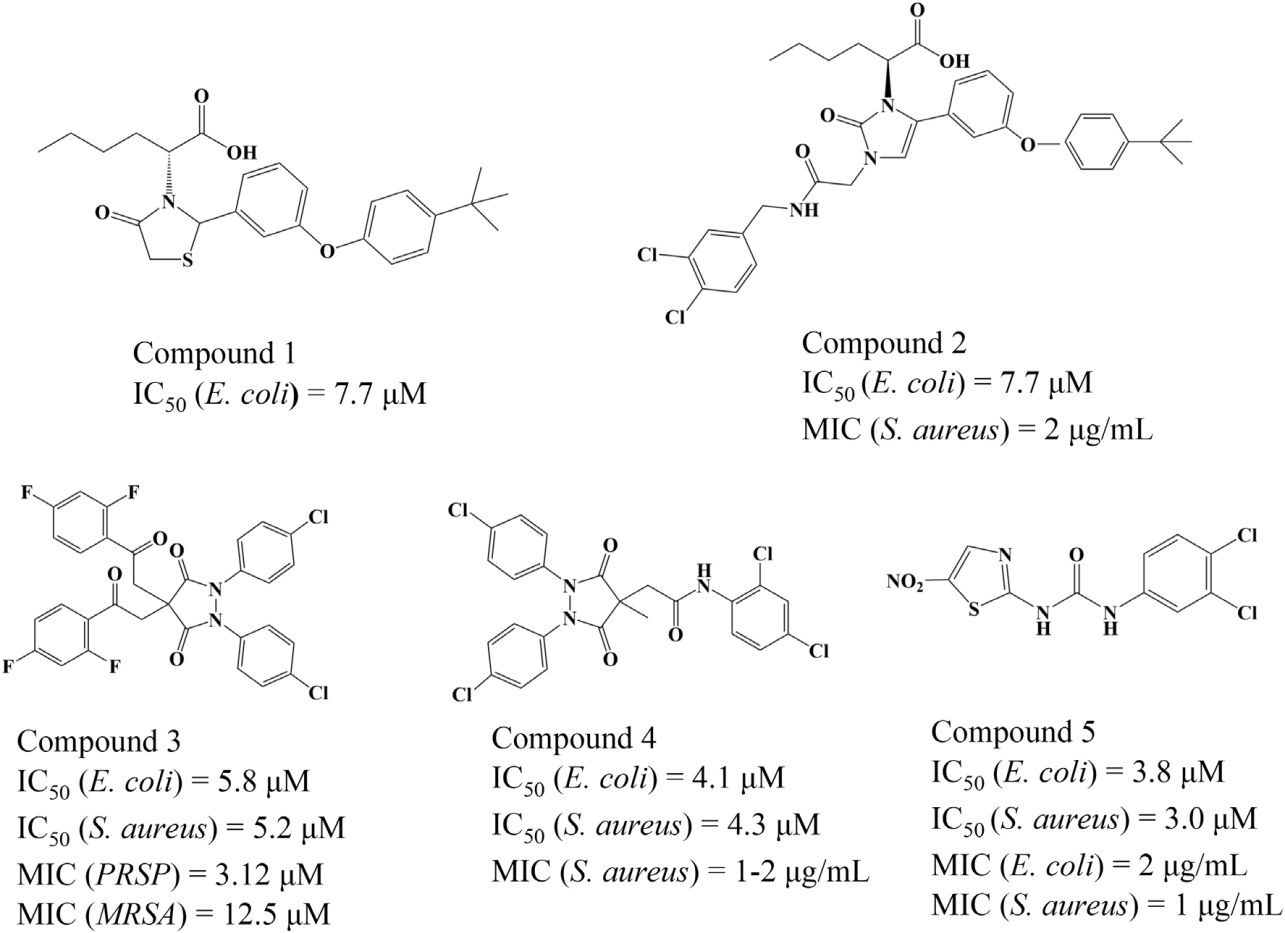
Chemical structures of reported MurB inhibitors.

Machine learning is a data*‐*driven approach that has been increasingly applied to drug discovery and development. AutoMolDesigner is a computational software tool that incorporates automated machine*‐*learning*‐*based activity and property prediction (Shen et al. 2024). In our previous study, AutoMolDesigner was used for machine*‐*learning*‐*based prediction to identify MurA inhibitors (Liu et al. 2025). In the present study, we used AutoMolDesigner (version 1.1) to construct five machine*‐*learning models. The model with the best predictive performance was subsequently selected to screen our in‐house compound library. Virtual screening is a computational strategy for prioritizing potential active compounds from large chemical libraries. Accordingly, the predicted candidates were further evaluated by molecular docking, with particular attention to their interactions with key catalytic residues in the MurB active site. Molecular clustering was subsequently performed to reduce structural redundancy and select representative compounds for further experimental evaluation.

The compounds identified through machine*‐*learning*‐*based virtual screening were subsequently evaluated for their inhibitory activity against recombinant *E. coli* MurB, leading to the identification of four active compounds, Q9, Q11, Q12, and Q13. Their cytotoxicity was then evaluated. To further characterize these active compounds, density functional theory (DFT) calculations were performed to investigate their electronic properties and molecular electrostatic potential distributions. Molecular dynamics (MD) simulations were subsequently conducted to investigate the stability and interaction patterns of the active compounds within the MurB binding site. Principal component analysis (PCA) was further performed to characterize the major conformational motions of the protein–ligand complexes, while MM*‐*GBSA calculations were used to estimate their binding free energies.

The overall workflow used in this study to discover MurB inhibitors is summarized in Figure 2. Collectively, these integrated approaches enabled the identification of potential MurB inhibitors and provided a foundation for further lead optimization and biological evaluation.

**FIGURE 2:**
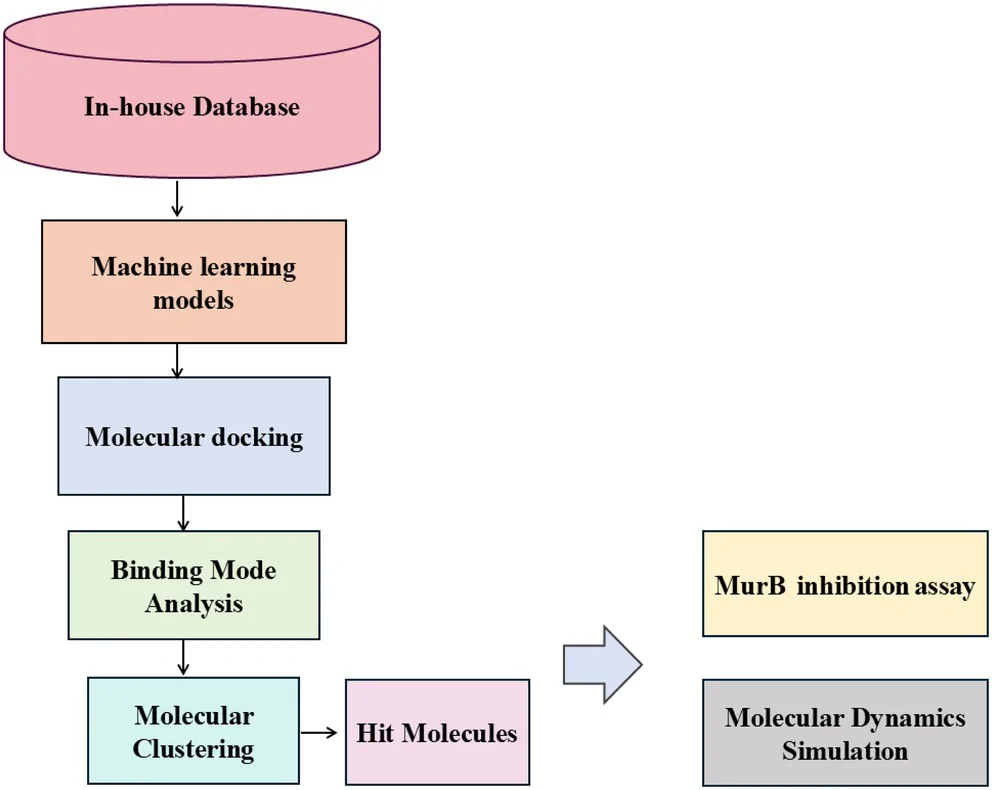
The flowchart for novel MurB inhibitor discovery.

## 2 Results and Discussion

### 2.1 Virtual Screening

#### 2.1.1 Dataset Compilation

A total of 112 *E. coli* MurB*‐*targeting inhibitors were retrieved from the ChEMBL database (https://www.ebi.ac.uk/chembl/). The dataset was preprocessed using the “Data Preparation” module of AutoMolDesigner, which standardized molecular structures, removed salts, and eliminated duplicates. After preprocessing, 84 unique inhibitors remained for subsequent analyses.

#### 2.1.2 Model Construction and Evaluation

The 84 MurB inhibitors were randomly divided, with 80% assigned to the training set and 20% to the test set. Five classification models were built using the “AutoGluon” module in AutoMolDesigner based on five molecular fingerprints/descriptors: RDKit_2D_Norm, RDKit_2D, ECFP_4, FCFP_6, and MACCS (Figure 3a). Model performance was assessed using true negative (TN), false positive (FP), false negative (FN), true positive (TP) (Heydarian et al. 2022), as well as Accuracy, Area Under the Receiver Operating Characteristic Curve (AUROC) (Kohn and Newman 2023), Matthews Correlation Coefficient (MCC) (Foody 2023), and F1_scores (Chicco and Jurman 2020).

**FIGURE 3:**
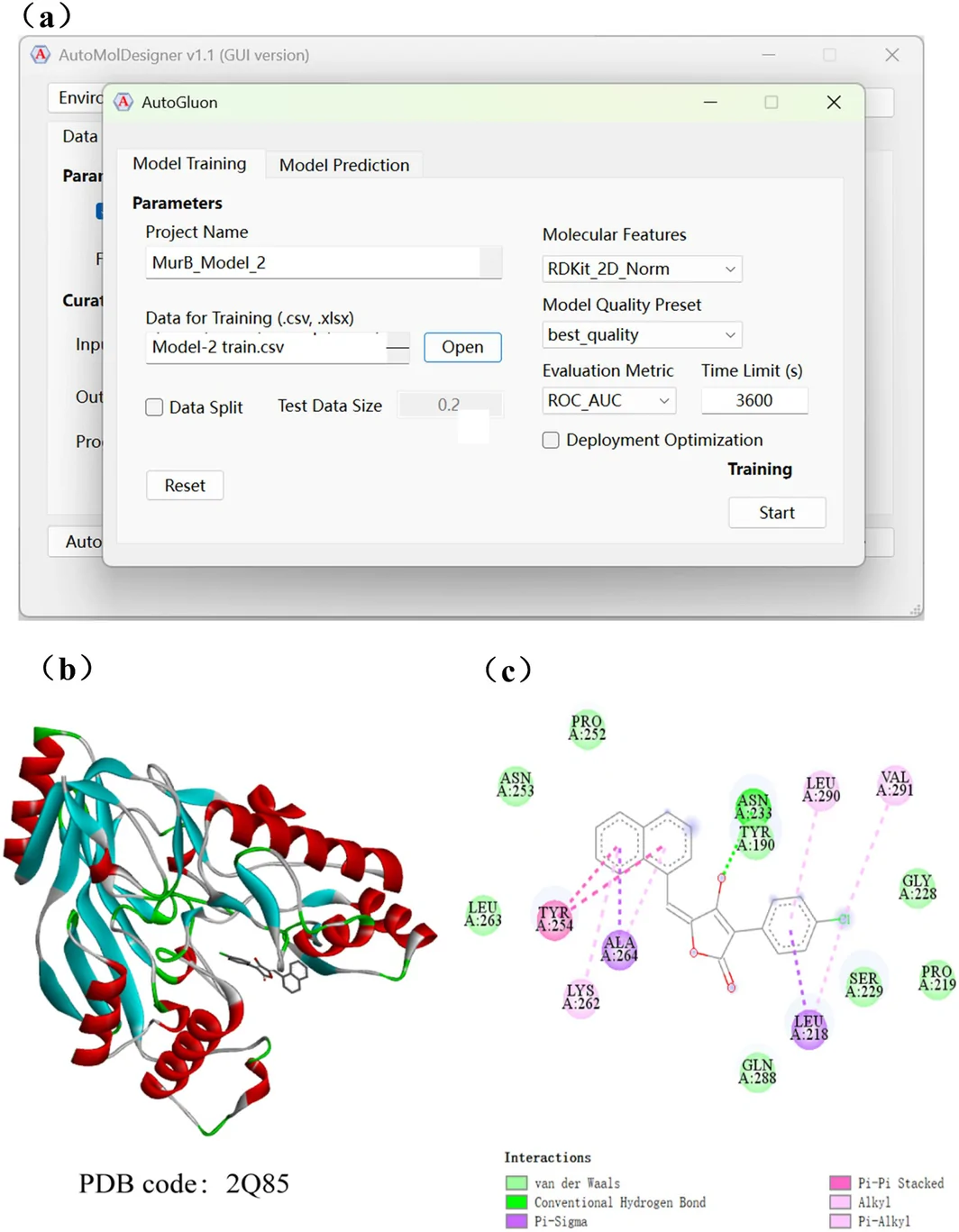
(a) AutoMolDesigner human*‐*computer interaction interface; (b) the crystal structure of 2Q85; (c) 2D diagram of ligand interactions with 2Q85.

Detailed metrics for each model on the test set are provided in Table 1. Based on predefined criteria (MCC > 0.5 and AUROC > 0.7), ECFP_4*‐*based Model_3 and MACCS*‐*based Model_5 were excluded due to low MCC values, and FCFP_6*‐*based Model_4 was excluded due to insufficient AUROC. Both RDKit_2D_Norm*‐*based Model_1 and RDKit_2D*‐*based Model_2 met the evaluation criteria, with Model_2 showing superior performance. Consequently, RDKit_2D*‐*based Model_2 was selected for subsequent virtual screening.

**TABLE 1:** Parameters of the five machine learning models.

Model	Fingerprint	TN	FP	FN	TP	Accuracy	AUROC	MCC	F1 score
Model_1	RDKit_2D_Norm	5	3	1	8	0.764	0.750	0.536	0.875
Model_2	RDKit_2D	5	3	1	8	0.764	0.777	0.536	0.857
Model_3	ECFP_4	4	4	2	7	0.647	0.583	0.290	0.763
Model_4	FCFP_6	5	3	1	8	0.764	0.652	0.536	0.824
Model_5	MACCS	4	4	2	7	0.647	0.583	0.290	0.753

#### 2.1.3 Molecular Docking

The screening procedure combined machine learning*‐*based filtering using RDKit_2D*‐*based Model_2, molecular docking with FRED, visual inspection of ligand binding conformations, and FCFP_6 fingerprint*‐*based clustering to ensure structural diversity.

Using Model_2, 238 compounds with predicted activity scores > 0.5 were identified from the internal library. Structural analysis of the *E. coli* MurB crystal structure (PDB: 2Q85) indicated that active ligands form hydrogen bonds with ASN A:233 (Figure 3b,c). Only compounds maintaining this interaction were retained. To reduce redundancy and maintain structural diversity, FCFP_6*‐*based clustering produced 16 representative clusters. Compounds were prioritized based on predicted activity, low FRED ChemGauss4 docking scores, and synthetic feasibility. Sixteen compounds meeting these criteria were selected for experimental validation (Table 2). PubChem (https://pubchem.ncbi.nlm.nih.gov/) searches confirmed they were previously unreported as MurB inhibitors.

**TABLE 2:** The 16 potential novel inhibitors and their inhibition rates against *E. coli* MurB at a concentration of 50 μM.

Name	ChemDiv ID number	Structure	Score	FRED Chemgauss4 score	%Inhibition mean ± SEM (50 μM)
Q1	AO*‐*364/37357113	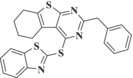	0.7338	*−*12.5951	18.27 ± 3.69
Q2	AK*‐*918/41675822	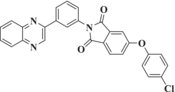	0.836	*−*11.4671	35.76 ± 4.48
Q3	AQ*‐*088/42014148	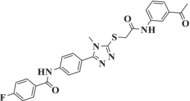	0.6053	*−*11.3774	17.28 ± 2.99
Q4	AN*‐*988/15131310	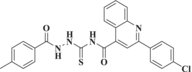	0.8115	*−*11.3542	21.90 ± 1.14
Q5	AE*‐*848/41828162	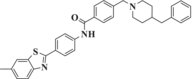	0.8958	*−*11.2777	34.31 ± 3.05
Q6	AQ*‐*088/41468920	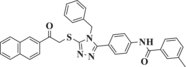	0.9363	*−*11.104	45.48 ± 2.63
Q7	AO*‐*476/43380303	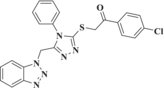	0.7005	*−*10.973	34.48 ± 2.19
Q8	AK*‐*968/12269883	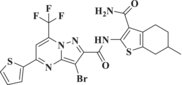	0.6413	*−*10.7809	30.99 ± 3.59
Q9	AN*‐*329/40034526	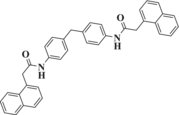	0.6949	*−*10.5472	60.30 ± 1.95
Q10	AG*‐*205/33663017	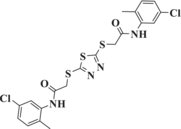	0.9516	*−*10.5309	22.26 ± 1.64
Q11	AN*‐*329/42100923	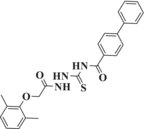	0.6234	*−*9.9816	52.23 ± 1.83
Q12	AG*‐*690/40124367	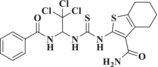	0.6131	*−*9.8949	65.68 ± 1.47
Q13	AK*‐*080/41414563	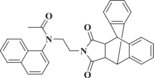	0.6110	*−*9.8842	59.05 ± 3.41
Q14	AG*‐*690/40751867	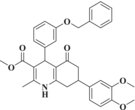	0.7518	*−*9.6361	43.94 ± 1.34
Q15	AK*‐*968/41024264	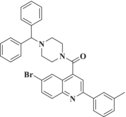	0.6503	*−*9.6255	42.54 ± 1.54
Q16	AN*‐*512/13661026	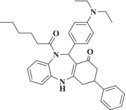	0.8937	*−*9.4393	48.62 ± 3.31

### 2.2 Identification of Hits

The recombinant plasmid pET*‐*28b*‐*MurB was synthesized and transformed into *E. coli* BL21(DE3) competent cells for MurB protein expression. SDS*‐*PAGE analysis showed a clear band, confirming successful purification (Figure S1). The 16 candidate compounds at 50 μM were evaluated for MurB inhibition activity (Table 2). Q9, Q11, Q12, and Q13 were identified as active inhibitors, showing inhibition rates of 60.30%, 52.23%, 65.68%, and 59.05%, respectively. The experimental hit rate of the virtual screening was 25%. Dose*–*response analysis yielded IC50 values of 34.44 ± 3.20 μM (Q9), 31.45 ± 3.44 μM (Q11), 25.08 ± 1.45 μM (Q12), and 25.94 ± 0.87 μM (Q13) (Figure 4).

**FIGURE 4:**
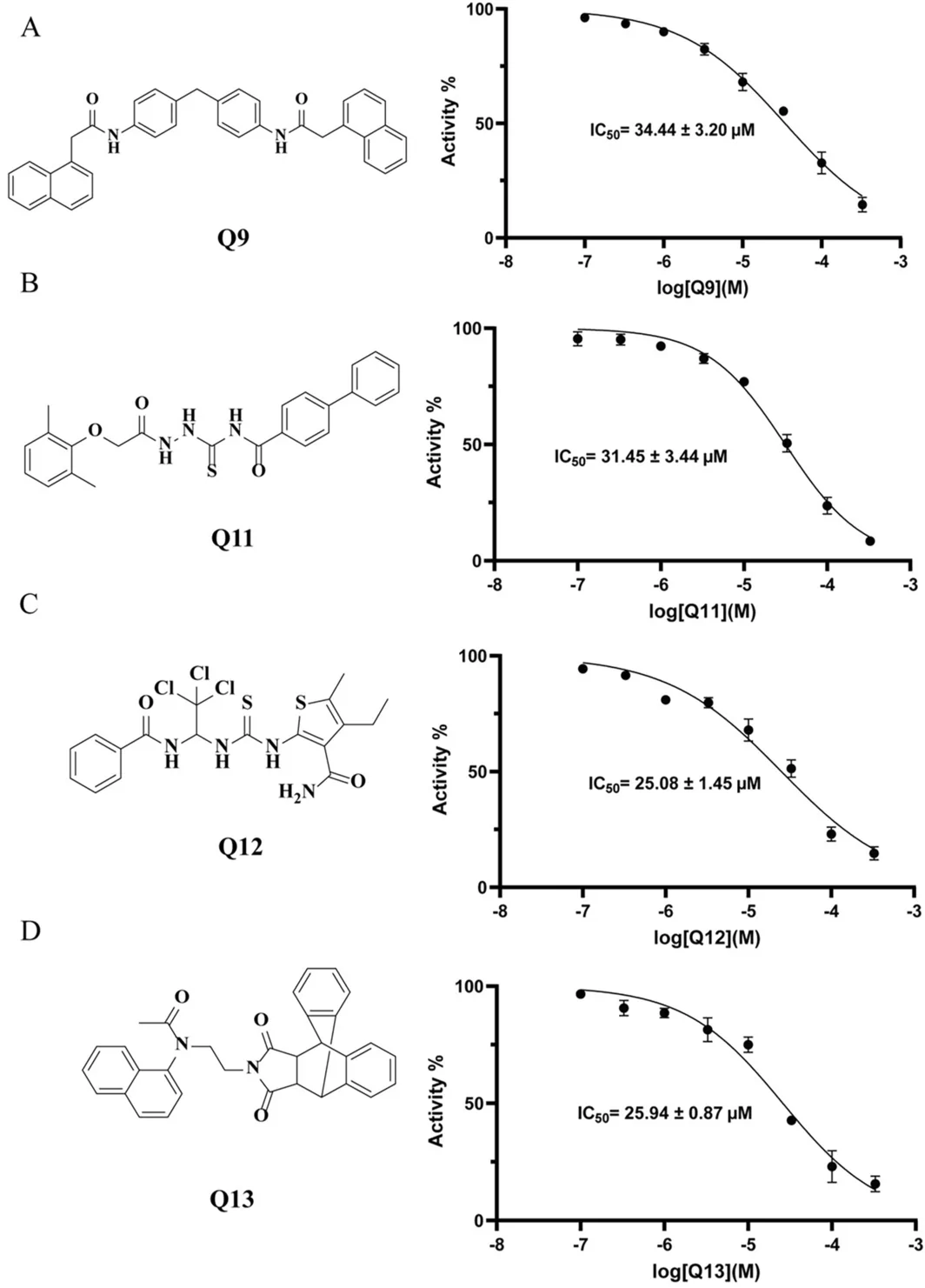
Dose*–*response curves and IC_50_ values of compounds (A) Q9, (B) Q11, (C) Q12, and (D) Q13 against *E. coli* MurB.

We further evaluated the antibacterial activity of Q9, Q11, Q12, and Q13 against *E. coli* ATCC 25922, and all four compounds showed MIC values greater than 64 μg/mL. Despite their inhibitory activity against recombinant MurB, none of the four compounds exhibited appreciable antibacterial activity.

The discrepancy between enzymatic MurB inhibition and whole‐cell antibacterial activity observed in the present study has also been reported for other MurB inhibitors. Yang et al. reported a series of 3,5‐dioxopyrazolidines that inhibited *E. coli* MurB but showed no activity against wild‐type *E. coli* . To test the role of outer membrane permeability, the authors further evaluated these compounds against an *E. coli* K‐12 imp strain with increased outer membrane permeability. However, only one compound showed activity against this strain, suggesting that increased outer membrane permeability alone may not be sufficient to restore whole‐cell activity (Yang et al. 2006). In a separate study, Gilbert et al. reported a series of 5‐hydroxy‐1H‐pyrazol‐3(2H)‐one derivatives with low‐micromolar inhibitory activity against *E. coli* MurB. Their structure–activity relationship analysis showed that improvements in MurB inhibitory potency and MIC values were correlated with increasing lipophilicity of the C‐4 substituent (Gilbert et al. 2006). These findings indicate that the relationship between structural features, MurB inhibitory potency, and whole‐cell antibacterial activity warrants further investigation. Future studies could systematically explore peripheral structural modifications and their relationships with cellular uptake, membrane permeability, and whole‐cell activity to guide the further optimization of MurB inhibitor candidates.

### 2.3 Cytotoxicity Evaluation

To evaluate the cytotoxicity of the compounds, the viability of HepG2 cells treated with Q9, Q11, Q12, and Q13 (0.1*–*100 μM) was determined using the CCK*‐*8 assay. As shown in Figure 5, most compounds exhibited limited cytotoxic effects at concentrations up to 33 μM. Q9 (Figure 5a) showed minimal effects on cell viability, with cell viability remaining above 80% throughout the tested concentration range. Similarly, Q13 (Figure 5d) showed a favorable cytocompatibility profile, maintaining cell viability above 80% up to 33 μM. Q11 (Figure 5b) caused a concentration*‐*dependent decrease in cell viability; however, the viability remained above approximately 60% even at the highest concentration tested (100 μM). Q12 (Figure 5c) showed limited effects on cell viability at concentrations up to 33 μM, whereas a decrease in cell viability was observed at the highest concentration tested (100 μM). Collectively, these results indicate that the evaluated MurB inhibitors exhibit relatively low cytotoxicity in HepG2 cells, providing support for their further biological evaluation.

**FIGURE 5:**
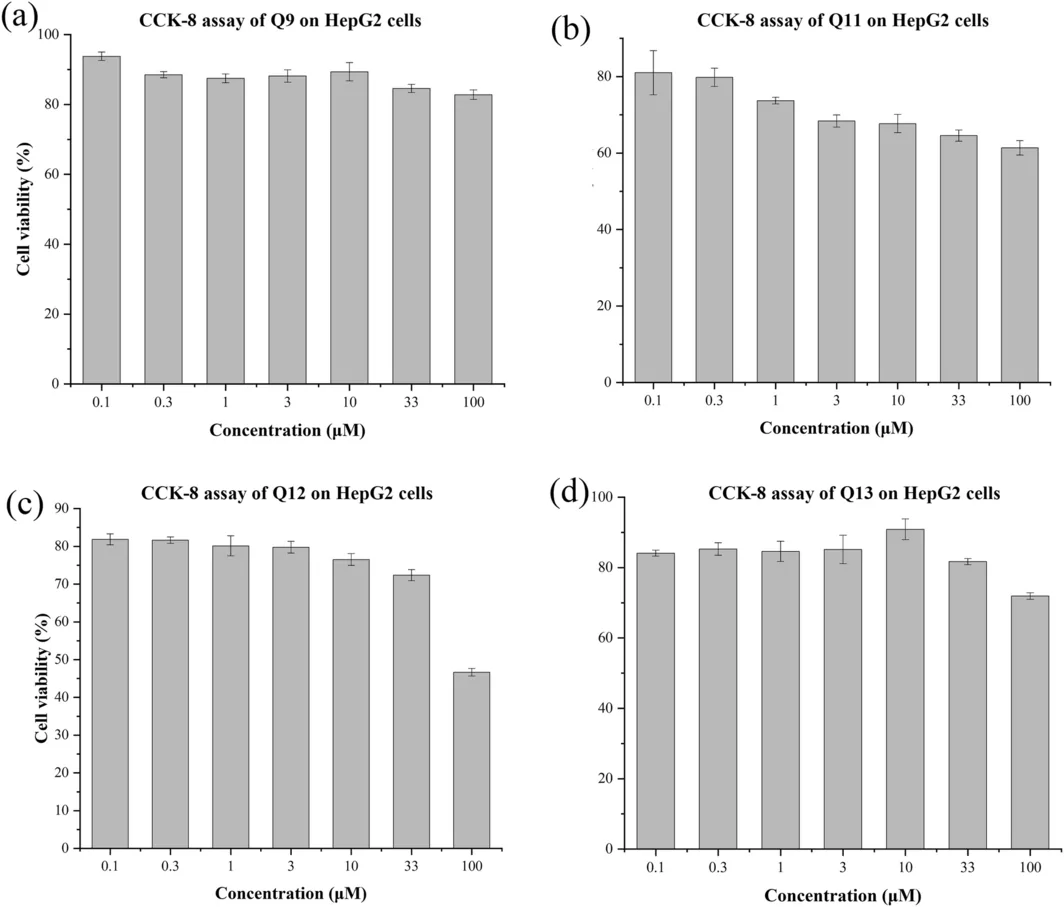
Cytotoxicity evaluation of Q9, Q11, Q12, and Q13 in HepG2 cells. (a) Q9; (b) Q11; (c) Q12; and (d) Q13.

### 2.4 Density Functional Theory Calculations

DFT calculations characterized the electronic structures of Q9, Q11, Q12, and Q13. The four compounds were fully optimized at the B3LYP/6*‐*31G(d,p) level using ORCA 6.0.1, followed by harmonic frequency calculations at the same level. No imaginary frequencies were observed for any of the optimized structures, confirming that the optimized geometries corresponded to local minima. The frontier molecular orbital energies and molecular electrostatic potential (MEP) distributions were subsequently evaluated.

The calculated frontier molecular orbital energies and HOMO–LUMO gaps are summarized in Table 3. Q11 had the highest HOMO energy (*−*5.5067 eV) and the lowest LUMO energy (*−*1.7520 eV), giving the smallest HOMO–LUMO gap (3.7547 eV). Q12 showed HOMO and LUMO energies of *−*5.6498 and *−*1.4518 eV, respectively, with a ΔE of 4.1980 eV. For Q9, the HOMO and LUMO energies were *−*5.6312 and *−*0.9894 eV, respectively, corresponding to a ΔE of 4.6418 eV. Q13 exhibited the lowest HOMO energy (*−*5.9427 eV) and a LUMO energy of *−*1.2682 eV, resulting in the largest ΔE (4.6745 eV). Accordingly, the calculated HOMO–LUMO gaps followed the order Q11 < Q12 < Q9 < Q13, demonstrating variation in the frontier orbital energy separation among the four compounds.

**TABLE 3:** Calculated frontier molecular orbital energies and HOMO‐LUMO gaps of Q9, Q11, Q12, and Q13.

Name	HOMO (eV)	LUMO (eV)	ΔE (eV)
Q9	*−*5.6312	*−*0.9894	4.6418
Q11	*−*5.5067	*−*1.7520	3.7547
Q12	*−*5.6498	*−*1.4518	4.1980
Q13	*−*5.9427	*−*1.2682	4.6745

The MEP distributions are shown in Figure 6. A common potential scale ranging from *−*0.008 to +0.008 a.u. was used for all four compounds to facilitate qualitative comparison. Q9 showed a distinct negative*‐*potential region associated with the conjugated framework, whereas relatively positive potential was localized around the oxygen*‐*containing moiety. Q11 exhibited the most pronounced separation between negative and positive potential, with negative potential distributed over electron*‐*rich regions and localized positive potential associated with the sulfur*‐* and oxygen*‐*containing moiety. Q12 displayed a more localized electrostatic pattern, with positive potential associated with its substituted framework and negative potential concentrated around the sulfur*‐* and halogen*‐*substituted region. Q13 showed negative potential across electron*‐*rich portions of the conjugated framework, whereas relatively positive potential was associated with the oxygen*‐*containing moiety. Overall, the MEP distributions revealed differences in the localization and spatial extent of positive and negative electrostatic potential among the four compounds, providing further electronic*‐*structure information for Q9, Q11, Q12, and Q13.

**FIGURE 6:**
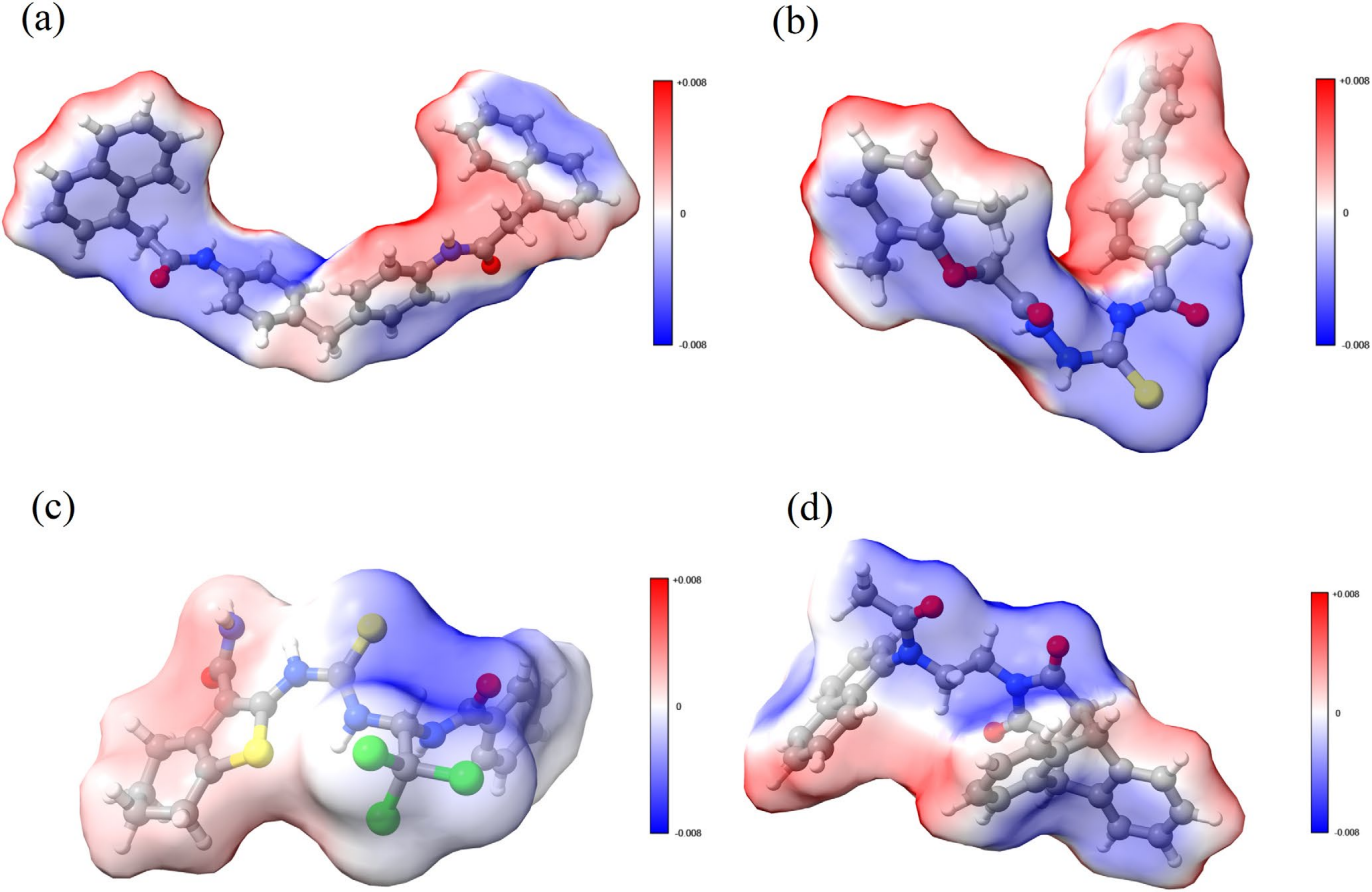
Molecular electrostatic potential maps of Q9, Q11, Q12, and Q13. (a) Q9, (b) Q11, (c) Q12, and (d) Q13.

### 2.5 Plausible Binding Modes From Molecular Dynamics Simulations

To investigate the binding stability and dynamic behaviors of different ligands in the *E. coli* MurB active site, 100 ns MD simulations were performed for the MurB*‐*co*‐*crystallized ligand complex and the Q9, Q11, Q12, and Q13*‐*MurB complexes. The co*‐*crystallized ligand complex served as a reference for identifying key ligand*‐*recognition features. The RMSD trajectory and two*‐*dimensional interaction diagram are shown in Figure S2.

As shown in Figure S2a, the MurB*‐*cocrystallized ligand complex exhibited slight fluctuations during the initial equilibration stage (0*–*10 ns), which may be attributed to the conformational adaptation of the ligand within the binding pocket. After approximately 35 ns, the protein backbone RMSD gradually stabilized and remained within a narrow fluctuation range throughout the remaining simulation period. Meanwhile, the ligand RMSD also maintained a relatively stable profile, suggesting that the co*‐*crystallized ligand could maintain a stable binding conformation within the active site. The representative equilibrated binding conformation was further extracted and analyzed (Figure S2b). The carbonyl oxygen of the co*‐*crystallized ligand formed a hydrogen bond interaction with TYR125, while its aromatic ring system established a π*‐*π stacking interaction with TYR254. These interactions may contribute to ligand recognition within the MurB active site and provide a structural reference for evaluating the binding modes of candidate compounds.

For the Q9*‐*MurB complex (Figure 7a), the RMSD trajectory showed moderate fluctuations during the initial simulation stage (0*–*5 ns), followed by gradual stabilization after approximately 60 ns. No obvious structural drift or ligand dissociation was observed throughout the simulation period, suggesting that Q9 could maintain a stable binding conformation within the MurB active site. The two*‐*dimensional interaction analysis (Figure 7c) demonstrated that the acetamide moiety of Q9 participated in hydrogen bond formation, in which the carbonyl oxygen formed a hydrogen bond interaction with SER229. Additionally, the aromatic ring system of Q9 established π*‐*π stacking interactions with TYR125 and TYR254. The interaction with TYR254 was consistent with the aromatic recognition feature observed in the co*‐*crystallized ligand, suggesting that Q9 retained an important structural characteristic involved in MurB recognition.

**FIGURE 7:**
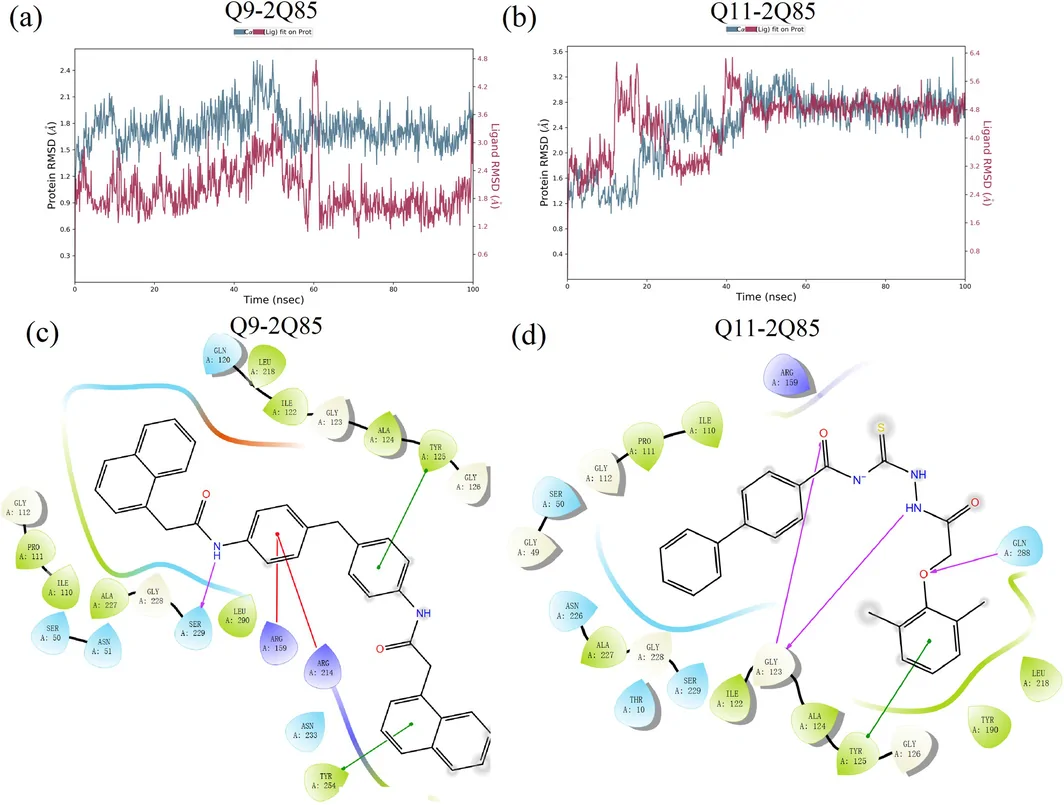
Molecular dynamics simulation results of Q9 and Q11 with *E. coli* MurB. (a) RMSD graph of Q9 with *E. coli* MurB in a 100 ns MD simulation; (b) RMSD graph of Q11 with *E. coli* MurB in a 100 ns MD simulation; (c) binding mode 2D graph of Q9; (d) binding mode 2D graph of Q11.

The Q11*‐*MurB complex (Figure 7b) displayed relatively larger fluctuations during the first 50 ns of simulation, suggesting a longer conformational adaptation process compared with Q9. However, the RMSD value gradually stabilized after 50 ns and remained within a relatively narrow fluctuation range during the subsequent simulation period, indicating the formation of a stable ligand*‐*protein complex. The interaction analysis (Figure 7d) revealed that the carbonyl oxygen of Q11 formed a hydrogen bond interaction with GLY123, while its aromatic ring system established a π*‐*π stacking interaction with TYR125. These interactions may contribute to maintaining the orientation of Q11 within the MurB binding cavity.

The Q12*‐*MurB complex (Figure 8a) exhibited slight fluctuations during the initial equilibration stage (0*–*10 ns), followed by a stable RMSD profile throughout the remaining simulation period. No obvious displacement of Q12 from the active site was observed during the simulation. According to the two*‐*dimensional interaction analysis (Figure 8c), the carbonyl oxygen of Q12 formed a hydrogen bond interaction with TYR125, while its aromatic ring system established a π*‐*π stacking interaction with TYR254. The conserved π*‐*π stacking interaction with TYR254 suggests that Q12 retained a similar aromatic recognition feature to that observed in the cocrystallized ligand.

**FIGURE 8:**
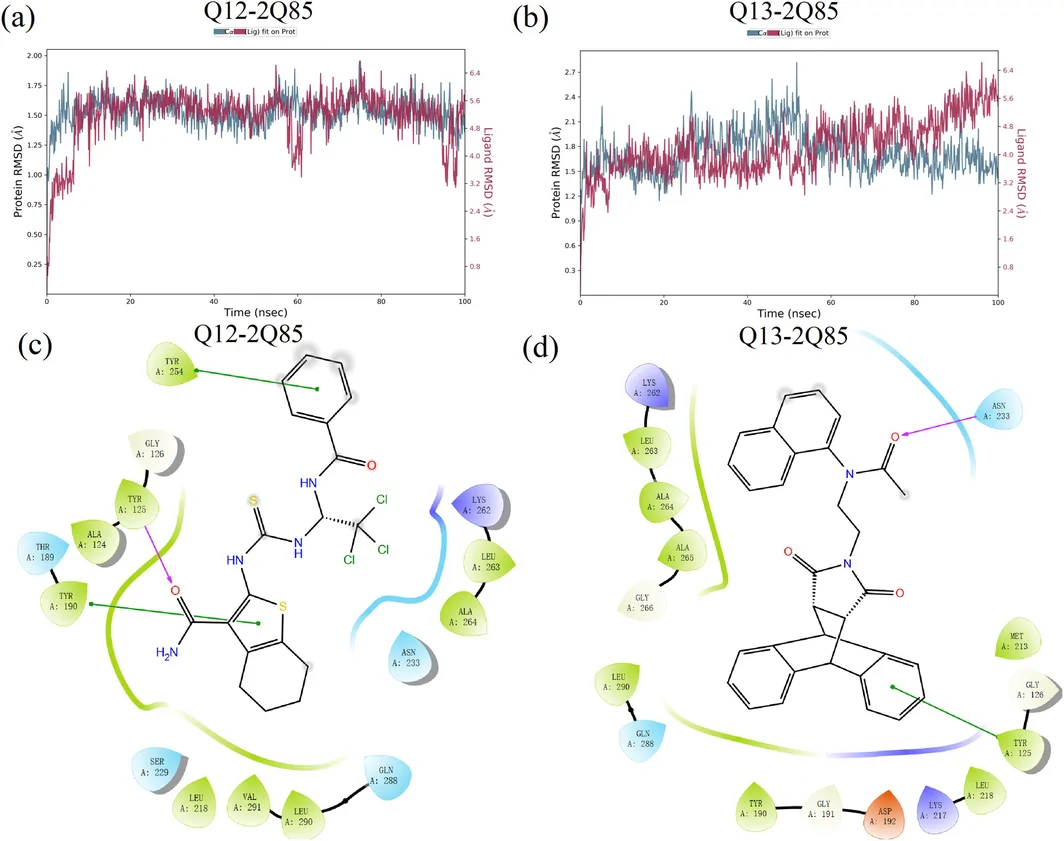
Molecular dynamics simulation results of Q12 and Q13 with *E. coli* MurB. (a) RMSD graph of Q12 with *E. coli* MurB in a 100 ns MD simulation; (b) RMSD graph of Q13 with *E. coli* MurB in a 100 ns MD simulation; (c) binding mode 2D graph of Q12; (d) binding mode 2D graph of Q13.

The Q13*‐*MurB complex (Figure 8b) underwent an initial conformational adjustment before reaching a stable equilibrium state. Although Q13 exhibited a certain degree of conformational flexibility during the simulation, no obvious departure from the active binding region was detected, suggesting that Q13 could maintain a stable association with MurB. The two*‐*dimensional interaction analysis (Figure 8d) showed that the carbonyl oxygen of Q13 formed a hydrogen bond interaction with ASN233, while its aromatic ring system established a π*‐*π stacking interaction with TYR125. Notably, the hydrogen bond interaction between the carbonyl oxygen of Q13 and ASN233 was consistent with the key interaction predicted by molecular docking. The persistence of this interaction during the MD simulation further supports the reliability of the docking*‐*based screening strategy. It provides structural support for the potential of Q13 as a MurB inhibitor candidate.

Overall, the MD simulations suggested that the cocrystallized ligand and Q9, Q11, Q12, and Q13 could maintain relatively stable binding conformations within the MurB active site. Conserved interactions involving TYR125 and TYR254 were observed in several candidate compounds, while the hydrogen*‐*bond interaction between Q13 and ASN233 may help maintain its binding mode with MurB.

### 2.6 MM *‐* GBSA Binding Free Energy Analysis

To further evaluate the binding affinities of the selected compounds toward MurB, MM*‐*GBSA calculations were performed using the equilibrated protein*‐*ligand complex structures obtained from molecular dynamics simulations. The calculated binding free energy Δ*G*_bind_ values are summarized in Table 4. The MM*‐*GBSA results showed that all four compounds exhibited favorable binding free energies, with Δ*G*_bind_ values ranging from *−*48.09 to *−*73.50 kcal/mol. Among them, Q9 showed the most favorable binding free energy (*−*73.50 kcal/mol), followed by Q13 (*−*70.45 kcal/mol), Q12 (*−*62.17 kcal/mol), and Q11 (*−*48.09 kcal/mol). The calculated binding free energy values indicated favorable interactions between Q9, Q11, Q12, and Q13 with MurB. These results further supported the potential binding stability of the selected compounds and provided additional computational evidence for their interactions with MurB.

**TABLE 4:** MM‐GBSA binding free energy calculations of Q9, Q11, Q12, and Q13.

Name	Δ*G*_bind_ (kcal/mol)
Q9	*−*73.50
Q11	*−*48.09
Q12	*−*62.17
Q13	*−*70.45

### 2.7 Principal Component Analysis

To further characterize the collective motions and conformational landscapes of MurB upon binding different inhibitors, PCA was performed based on the MD trajectories of the Q9*‐*, Q11*‐*, Q12*‐*, and Q13*‐*bound complexes. The cumulative contributions of the first three principal components (PC1–PC3) were 37.50%, 46.25%, 18.95%, and 30.15% for the Q9*‐*, Q11*‐*, Q12*‐*, and Q13*‐*bound complexes, respectively (Figure 9). These results indicated that the major collective motions were distributed differently among the inhibitor*‐*bound systems, reflecting ligand*‐*dependent variations in the conformational dynamics of MurB.

**FIGURE 9:**
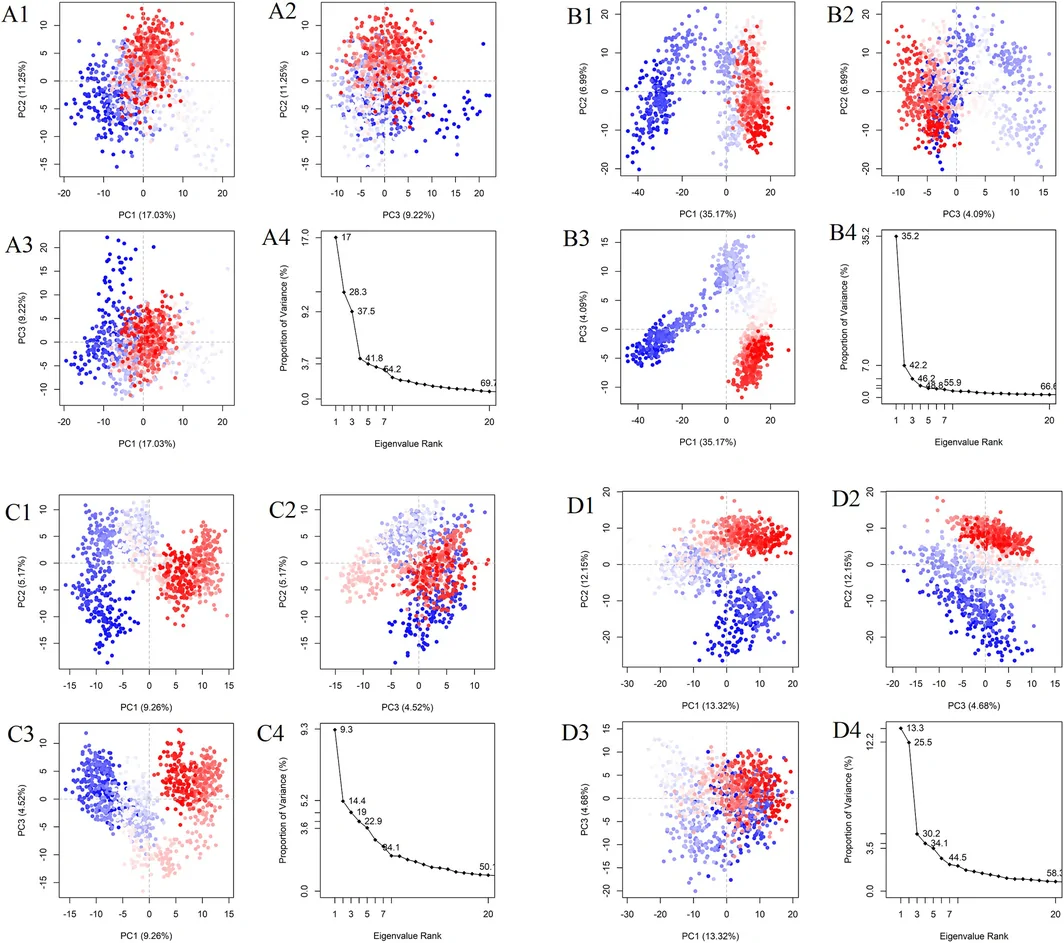
PCA results of inhibitor*‐*bound complexes. (A) Q9*‐*bound complex, (B) Q11*‐*bound complex, (C) Q12*‐*bound complex, and (D) Q13*‐*bound complex. Panels 1*–*3 show the PC1*‐*PC2, PC2*‐*PC3, and PC3*‐*PC1 projections, respectively, and panel 4 shows the eigenvalue distribution.

The PCA projections revealed distinct conformational distributions among the four inhibitor*‐*bound complexes. The Q9*‐*bound complex exhibited a relatively compact and continuous distribution in the PC1*‐*PC2, PC2*‐*PC3, and PC3*‐*PC1 spaces, with considerable overlap among sampled conformations, suggesting that this system mainly remained within a similar conformational ensemble during the simulation. In contrast, the Q11*‐*bound complex showed a pronounced separation into two distinct clusters, accompanied by the highest PC1 contribution (35.17%), indicating that this system sampled clearly different conformational regions. The Q12*‐*bound complex displayed a more dispersed distribution with multiple populated regions, suggesting a broader conformational sampling behavior. Meanwhile, the Q13*‐*bound complex exhibited two partially overlapping clusters, representing an intermediate conformational distribution between the compact ensemble observed for Q9 and the separated states observed for Q11.

Overall, PCA demonstrated that different inhibitors modulated the global motions and conformational landscapes of MurB in distinct ways. The Q11*‐*bound complex showed the most prominent conformational separation, whereas the Q9*‐*bound complex maintained a relatively continuous and restricted conformational distribution. The Q12*‐* and Q13*‐*bound complexes exhibited moderate conformational variability, indicating different degrees of conformational adaptation upon inhibitor binding. These findings provide further insights into the influence of inhibitor binding on the collective motions and conformational diversity of MurB.

## 3 Conclusion

In this study, an integrated strategy combining machine learning‐based virtual screening and experimental validation was established to identify potential *E. coli* MurB inhibitors. We identified four compounds, Q9, Q11, Q12, and Q13, with IC₅₀ values ranging from 25.08 to 34.44 μM. Cytotoxicity evaluation indicated low cytotoxicity of these compounds, while DFT, MD, PCA, and MM‐GBSA analyses provided complementary molecular‐level insights into their electronic properties, binding interactions, and conformational behavior. However, the identified compounds showed limited antibacterial activity. Future studies should focus on systematic structure–activity relationship studies to guide their further optimization. In addition, the limited size and chemical diversity of the in‐house compound library may have constrained the chemical space explored; screening larger and more diverse compound libraries may facilitate the discovery of additional MurB inhibitor scaffolds. Furthermore, more systematic prioritization of candidate compounds after molecular clustering may improve the efficiency of selecting structurally diverse and biologically relevant candidates.

## 4 Materials and Methods

### 4.1 Machine Learning

#### 4.1.1 Data Preparation and Processing

A total of 112 small*‐*molecule compounds with reported MurB inhibitory activity (IC_50_ values) were collected from the ChEMBL database using *E. coli* MurB as the search criterion. The “Data Preparation” module in AutoMolDesigner was applied to standardize and neutralize the SMILES representations of all compounds. According to Lipinski's rule (Lipinski 2012), compounds with a molecular weight below 600 Da and fewer than 20 rotatable bonds were retained for further analysis. Compounds were subsequently classified as active or inactive based on whether their IC₅₀ values were below 50 μM. The structural diversity of the dataset was evaluated to ensure sufficient chemical variety among the selected compounds.

#### 4.1.2 Model Construction and Training

Machine learning models were built using the “AutoGluon” module in AutoMolDesigner. Initially, 80% of the dataset was randomly assigned to the training set, while the remaining 20% was reserved as the testing set through the module's “Data Split” function. Model training and prediction were performed using the “Model Training” and “Model Prediction” modules, respectively. In the “Model Training” module, molecular descriptors including RDKit_2D_Norm, RDKit_2D, ECFP_4, FCFP_6, and MACCS were used to encode the compounds. The “Model Quality Preset” was set to “best quality”, and the “Evaluation Metric” for classification was defined as ROC_AUC. All other parameters were kept as the default.

#### 4.1.3 Model Performance Evaluation

The performance of the trained models was evaluated using multiple metrics, including confusion matrix, accuracy, area under the ROC curve (AUC), Matthews correlation coefficient (MCC), and F1*‐*score. The confusion matrix contained four elements: true negative (TN), false positive (FP), false negative (FN), and true positive (TP). TN represents correctly predicted inactive compounds, FP denotes inactive compounds incorrectly predicted as active, FN corresponds to active compounds incorrectly predicted as inactive, and TP indicates correctly predicted active compounds.

### 4.2 The Method of Virtual Screening

#### 4.2.1 ML Model Building

An in*‐*house compound library was employed for virtual screening of MurB inhibitors. This library comprised 16,828 compounds, including commercially available compounds from the Specs library, high*‐*throughput screening compounds from the ChemDiv library, and compounds synthesized in our laboratory. All molecules were standardized using the “Data Preparation” module in AutoMolDesigner. In the “Molecular Features” section of the “Model Prediction” module, each compound was represented by a specific molecular descriptor or fingerprint. The selected descriptor/fingerprint was applied to the ML model with the best performance for prediction. Compounds with ML scores higher than 0.5 were retained and subsequently prepared using the “Prepare Ligands” module in Discovery Studio.

#### 4.2.2 Molecular Docking

The MurB protein structure was retrieved from the Protein Data Bank (PDB, https://www.rcsb.org), and the structure (PDB ID: 2Q85) was preprocessed using the “Prepare Protein” protocol in the “Macromolecules” module of Discovery Studio. Preprocessing included the removal of water molecules and heteroatoms, followed by structure optimization. Candidate ligands were processed with the “Prepare Ligands” module in Discovery Studio to generate their protonation states at pH 7.4 and all possible stereoisomers. Docking was performed using the FRED module in OpenEye, with the atomic coordinate center of the co*‐*crystallized ligand defined as the docking center and an active site radius of 8 Å. Docking poses were ranked using the FRED ChemGauss4 scoring function, where lower scores indicate stronger predicted binding affinity. For each compound, the top five highest*‐*scoring conformations were retained. Docked conformations were visually inspected, and compounds forming hydrogen bond interactions with the key residue ASN A:233 were selected. To further enhance structural diversity, selected compounds were clustered using the “Cluster Ligands” module in Discovery Studio based on FCFP_6 fingerprints, and representative compounds from each cluster were chosen. Structural searches were then conducted in PubChem to verify that the selected compounds had not been previously reported as MurB inhibitors.

#### 4.2.3 Density Functional Theory Calculations

We performed density functional theory calculations using ORCA 6.0.1 (Neese 2025). We fully optimized the structures of Q9, Q11, Q12, and Q13 at the B3LYP/6*‐*31G(d,p) level of theory, followed by harmonic frequency calculations at the same level. No imaginary frequencies were observed for any of the optimized structures, confirming that the optimized geometries corresponded to local minima on the potential energy surface. The energies of the highest occupied molecular orbital (HOMO) and lowest unoccupied molecular orbital (LUMO) were obtained from the optimized structures, and the HOMO*‐*LUMO energy gap (ΔE) was calculated as the difference between the LUMO and HOMO energies. Molecular electrostatic potentials were calculated from the optimized wavefunctions using the orca_vpot utility in ORCA 6.0.1. The resulting MEP maps were visualized on the molecular surfaces using UCSF ChimeraX version 1.12 (Pettersen et al. 2021).

#### 4.2.4 Molecular Dynamics Simulation

MD simulations of MurB protein*‐*ligand complexes were conducted using the Desmond module in the Schrödinger software suite. We first built the complex systems with the “System Builder” module and solvated them in an orthorhombic water box using the TIP4P water model. The water box boundaries were set 10 Å from the outer surface of the protein to adequately mimic the solvent environment under physiological conditions. We assigned atomic force field parameters using the OPLS4 force field and added appropriate counterions to neutralize the system. Prior to production runs, the system underwent energy minimization and a 300*‐*ps Brownian dynamics simulation for structural equilibration. The system was then gradually heated from 0 to 310 K under the NVT ensemble, maintaining a temperature of 310 K. Subsequently, 200*‐*ns molecular dynamics simulations were performed under constant temperature and pressure conditions (310 K and 1.01 bar) using Desmond. Trajectories were saved every 100 ps for subsequent analyses. Protein*‐*ligand interactions were examined after simulations using Schrödinger's Simulation Interaction Diagram tool, and root*‐*mean*‐*square deviations (RMSD) of both protein and ligand were calculated to assess conformational stability.

#### 4.2.5 MM *‐* GBSA Binding Free Energy Analysis

To further evaluate the binding affinities of the selected protein*‐*ligand complexes, MM*‐*GBSA calculations were performed using the Prime MM*‐*GBSA module implemented in the Schrödinger molecular modeling suite (Release 2021*‐*2). The equilibrated protein*‐*ligand complex conformations obtained from molecular dynamics simulations of Q9, Q11, Q12, and Q13 were used as the initial structures for binding free energy calculations. During the calculations, amino acid residues within 3 Å of the ligand*‐*binding site were treated as flexible to account for local structural adaptation. The binding free energy (Δ*G*_bind_) was calculated according to the following equation:

(1) $$∆G_{\text{bind}}=G_{\text{compler}}-\left(G_{\text{protein}}+G_{\text{ligand}}\right)$$

where *G*_complex_, *G*_protein_, *and G*_ligand_ represent the free energies of the protein*‐*ligand complex, isolated protein, and isolated ligand, respectively. The calculations were performed using the OPLS4 force field combined with the VSGB implicit solvent model. The calculated Δ*G*_bind_ values were used to compare the relative binding affinities of Q9, Q11, Q12, and Q13 toward the target protein. More negative Δ*G*_bind_ values indicate stronger and more favorable interactions between the ligand and receptor.

#### 4.2.6 Principal Component Analysis

PCA was performed to characterize the conformational dynamics of protein*‐*ligand complexes based on the MD trajectories. The trajectories were extracted from the simulations and converted into DCD format for subsequent analysis using the Bio3D package implemented in R.

For each complex, trajectory and reference structure files were loaded using the read.dcd and read.pdb functions, respectively. The Cα atoms of the protein were selected using the atom.select function. To eliminate overall translational and rotational motions, all trajectory frames were aligned to the reference structure through least*‐*squares fitting using the fit.xyz function. PCA was then performed using the pca.xyz function based on the aligned Cα coordinates. The covariance matrix was calculated to obtain the principal components and their corresponding eigenvalues. The projections of trajectories onto PC1, PC2, and PC3 were further analyzed to evaluate the major collective motions and conformational distributions of different protein*‐*ligand complexes.

### 4.3 In Vitro Enzymatic Evaluation

#### 4.3.1 MurB Protein Expression and Purification

The amino acid sequence of *E. coli* MurB was retrieved from the UniProt database (https://www.uniprot.org/). The *E. coli* MurB gene was synthesized with BamHI and HindIII restriction sites and cloned into the pET*‐*28b(+) vector containing an N*‐*terminal 6 × His tag for affinity purification. The recombinant plasmid was synthesized and constructed by GenScript Biotech Corporation. The plasmid was transformed into *E. coli coli* BL21 (DE3) competent cells via heat shock. Transformed cells were incubated in a shaking incubator at 37°C for 1 h, plated on LB agar containing kanamycin, and incubated overnight. Single colonies were inoculated into LB medium with kanamycin and cultured overnight at 37°C with shaking at 200 rpm. The overnight culture was diluted 1:100 into 500 mL of fresh LB medium with kanamycin and incubated at 37°C, 200 rpm, until OD600 reached 0.6*–*0.8. Protein expression was induced with 0.5 mM IPTG at 25°C for 8 h. Cells were harvested by centrifugation at 4900 rpm, 4°C for 40 min, resuspended in lysis buffer (50 mM Tris*–*HCl, 100 mM NaCl, 1 mM EDTA, 5% glycerol, pH 8.0), and lysed by ultrasonication on ice until the suspension became clear. The lysate was centrifuged at 25,000 rpm, 4°C for 1 h, and the supernatant was collected. It was applied to a pre*‐*equilibrated Ni^2+^ *‐*NTA affinity column, incubated for 1 h at room temperature, and eluted with buffer containing 250 mM imidazole. Fractions containing *E. coli* MurB were collected, and purity was confirmed by SDS*‐*PAGE. Protein concentration was determined, and 10 mL of purified protein (1.6 mg/mL) was aliquoted and stored at *−*80°C for subsequent experiments.

#### 4.3.2 MurB Enzymatic Activity and Inhibition Assay

MurB enzymatic activity was measured using the NADPH*‐*dependent reduction of UNAGEP, which is generated from UNAG and PEP by MurB (Shapiro et al. 2012). The decrease in absorbance at 340 nm was monitored to quantify NADPH consumption, reflecting MurB activity. Compounds were prepared as 100 mM stock solutions in DMSO and tested at various concentrations.

Each 96*‐*well plate reaction contained water, 50 mM Tris*–*HCl, 0.2 mM UNAG, 0.2 mM PEP, 500 nM MurA, 5 μM MurB, 0.5 mM NADPH, and 10 mM KCl. Reactions were performed at 37°C, and absorbance at 340 nm was recorded. Protein*‐*free wells served as negative controls, and DMSO controls were included to exclude solvent interference. IC_50_ values were calculated from dose*–*response curves using GraphPad Prism. All experiments were repeated independently three times, and results are reported as mean ± SEM.

#### 4.3.3 In Vitro Antibacterial Activity Assay

The in vitro antibacterial activity of the four screened compounds was evaluated against *E. coli* ATCC 25922 using the broth microdilution method. Minimum inhibitory concentrations (MICs) were determined by twofold serial dilution, with levofloxacin serving as the reference antibiotic. The compounds were dissolved in dimethyl sulfoxide (DMSO) to prepare stock solutions at 3.2 mg/mL. An *E. coli* suspension adjusted to 0.5 McFarland turbidity was diluted with liquid LB medium to obtain a final inoculum of approximately 10^5^ CFU/mL.

For the assay, 196 μL of the bacterial suspension was added to the wells of the first column (A1‐H1) of a sterile 96‐well microplate, while 100 μL of liquid LB medium was added to the remaining wells (A2‐H12). Subsequently, 4 μL of the compound stock solution was added to the first column and mixed thoroughly. A 100‐μL aliquot was then transferred sequentially from each column to the next, with thorough mixing at each step, to establish a twofold serial dilution across columns 1–12. Finally, 100 μL was removed from the wells of column 12 and discarded, resulting in a final volume of 100 μL per well. The final compound concentrations ranged from 64 to 0.03125 μg/mL.

The plates were incubated at 37°C for 17–20 h. Bacterial growth was assessed visually based on turbidity. The MIC was defined as the lowest compound concentration at which no visible bacterial growth was observed. All experiments were performed in triplicate. Wells containing LB medium alone served as the blank control, and levofloxacin was included as the positive control.

### 4.4 Cytotoxicity Assay

The cytotoxic effects of the selected compounds on HepG2 cells were evaluated using a Cell Counting Kit*‐*8 (CCK*‐*8) assay. HepG2 cells were seeded into 96*‐*well plates and cultured at 37°C under 5% CO_2_ conditions for 24 h to allow cell attachment. Subsequently, cells were treated with compounds Q9, Q11, Q12, and Q13 at final concentrations of 0.1, 0.3, 1, 3, 10, 33, and 100 μM, respectively, and incubated for an additional 24 h. After treatment, CCK*‐*8 reagent was added to each well, and the cells were incubated according to the manufacturer's instructions. The absorbance at 450 nm was measured using a microplate reader. Cell viability was expressed as a percentage relative to the untreated control group.

## Author Contributions

**Shuhua Song:** writing – original draft, formal analysis. **Qingxin Liu:** methodology, software, data curation. **Aoqi Luo:** data curation, formal analysis. **Xiao Shi:** conceptualization, supervision. **Xinxin Si:** conceptualization, writing – review and editing, project administration, supervision.

## Funding

The Talent Introduction Startup Fund of Jiangsu Ocean University (Project No. KHJ2021000124) supported this research.

## Conflicts of Interest

The authors declare no conflicts of interest.

## Supporting information


**Figure S1:** Purified *E. coli* MurB protein.
**Figure S2:** Molecular dynamics simulation and 2D interaction analysis of the *E. coli* MurB‐cocrystallized ligand complex (PDB ID: 2Q85). (a) RMSD profile during a 100 ns MD simulation; (b) 2D interaction diagram of the co‐crystallized ligand in the active site of *E. coli* MurB.

## Data Availability

The data supporting the findings of this study are available in the article's Supplementary Material.
