# Supplementary material for: Machine Learning‐Guided Discovery of Novel Inhibitors of Escherichia coli MurB
Source: Chem Biol Drug Des. 2026 Nov 5;108(5):e70422. doi: 10.1111/cbdd.70422 (PMC13735866; doi:10.1111/cbdd.70422)
Supplement: Supplementary file 1 [file CBDD-108-e70422-s001.docx]

**Machine Learning-Guided Discovery of Novel Inhibitors of *Escherichia coli* MurB**

Shuhua Song, Qingxin Liu, Aoqi Luo, Xiao Shi, Xinxin Si^*^

*School of Pharmacy, Jiangsu Ocean University, Lianyungang, Jiangsu 222005, China;*

Corresponding author.

E-mail: sixx@jou.edu.cn (Xinxin Si)

Figure S1 Purified *E. coli* MurB protein

Figure S2. Molecular dynamics simulation and 2D interaction analysis of the *E. coli* MurB-cocrystallized ligand complex (PDB ID: 2Q85). (a) RMSD profile during a 100 ns MD simulation; (b) 2D interaction diagram of the co-crystallized ligand in the active site of E. coli MurB.
